# Supernumerary Dens Sapientiæ

**Published:** 1853-10

**Authors:** J. Richardson

**Affiliations:** Cincinnati


*Supernumerary Dens Sepientiæ.* By J. RICHARDSON, D. D. S., Cincinnati.

The following case, though perhaps not altogother anomalous, is sufficiently rare, I think, to entitle it to publicity. No marked departure from the ordinary or natural developement of the teeth, can be regarded with entire indifference, when a knowledge of it may prove either curious or instructive to the dentist, or advantageous to the patient. Such aberrations, always possesses an historical as well as practical interest, and as the aggregate, experience of the profession is dependent for its completeness upon the personal observations and contributions of its several members; it becomes at once, a duty, if not a pleasure, on their part, to contribute to this general fund, whatever is really valuable, either from its novelty or practical tendency.

Some three or four weeks since, a gentleman, about thirty years of age, called at my office, and requested me to examine the left dens sapientiæ of the lower jaw, which he said had been exceedingly painful for some days. On examination, I found the crown of the tooth perfectly round, quite large, and somewhat crowded into the angle of the jaw. The gums, as is usual in such cases, were much swollen, and highly inflamed, and a considerable portion, posterior to the teeth, destroyed by ulceration. The inflammation had extended to the fauces, producing some soreness of throat, and a slight hacking cough.

I advised the removal of the tooth, as the means best calculated to afford him speedy and permanent relief, to which he assented. After its extraction, I recommended the use of the solid nitrate of silver, to the ulcerated portion, in conjunction with an astringent wash. This tooth had four well-defined roots.

In six or eight days, he returned. The gums were entirely restored to health, and he expressed himself highly gratified with the result of the operation. He informed me however, that soon after the loss of this tooth, the gums around the dens sapientiæ of the opposite side, of the same jaw, became in like manner involved, and ran a course similar to the first. Its extraction was determined upon, and after making a free incision through a flap of gum covering the posterior half of the crown, I removed it without difficulty, but with considerable pain to the patient, whose gums were so sensitive, that he could scarcely tolerate the most careful examination. The crown of this tooth was also, unusually large, and had four fangs, but not so distinctly marked as the former. There was the usual complement of teeth in front of these, well developed, and well arranged. I dismissed my patient with confident assurances of entire relief, in a few days. The sequel will test the correctness of my prognosis.

A few days since, he again called, and expressed some disappointment, at not having realized the same relief from the last, that he had fiom the former operation, and that the parts did not seem disposed to heal kindly. He complained of a sensation of decided discomfort, and pain at the bottom of the cavity, with soreness about the angle of the jaw, extending into the temple and neck. These sensations, he said, had been preceded for some days, by a highly painful condition of the muscles, associated with the inferior maxilla, rendering every motion of the jaw exceedingly painful. Also a severe and deep-seated pain under the molar bone, extending from its anterior margin to the ear, which was aggravated every ten or fifteen minutes, into sharp lancinating flashes of pain, of a most distressing character.

He was of the opinion, that the diseased action about the tooth, had extended to the bottom of the socket, and was involving the maxillar, nothwithstanding he had made dilligent use of the means recommended.

On examination, I found the gums entirely healthy, with the exception of some slight degree of tumefaction, with some tenderness to the touch, but no ulceration visible. The cavity however, remained open, and manifested no disposition to close up, and obliterate, as that upon the opposite side had previously done. On introducing a probe into the socket, which was quite deep, and as large as the crown of the extracted tooth, I could distinctly feel at the bottom, a hard, smooth substance, which I took at first, to be a portion of denuded process, or jaw bone, exposed by the ulceration having extended itself in that direction. But there was a *smoothness* about the surface, and a peculiar *click*, when struck by the instrument, that was suggestive of something, having a finer *polish*, than that usually bestowed on ulcerated jaw bone, and smacked wonderously of enamel. On a more critical examination with the mouth glass, and suitable instruments, I discovered deeply imbedded in the jaw, not what we had at first suspected, but what proved to be the grinding surface of an apparently well developed crown, of a second wisdom tooth, occupying a position directly underneath the one extracted. It was some five or six lines beneath the free margin of the gums; was, so far as I could determine, about the size of the one removed; and will, if it advances, occupy precisely the same position in the jaw.

The probabilities are, that there is little of this tooth but the crown, and that the fangs, if developed at all, are but imperfectly so. There are no conditions on the opposite side of the jaw, that at present indicate the existence of a supernumerary tooth, upon that side.

I would only add, that during the progress of these morbid conditions, the patient's general health, has been seriously compromised. Although constitutionally robust, it was with difficulty, he could get to and from the office. The continued draught upon the energies of the nervous system, by the persistent and inveterate pain which he suffered, and by loss of sleep, resulted in extreme debility; loss of appetite, colliquative night-sweats, and a train of irregular nervous symptoms, peculiarly harassing. All these conditions, however, have been materially mitigated, by the extraction of the teeth.

